# Exploring gender differences in empathy development among medical students: a qualitative analysis of reflections on juvenile correctional school visits

**DOI:** 10.1080/10872981.2025.2500556

**Published:** 2025-05-04

**Authors:** Hsiang-Chin Hsu, Tzu-Ching Sung

**Affiliations:** a Department of Emergency Medicine, National Cheng Kung University Hospital, Tainan, Taiwan; b School of Medicine, College of Medicine, National Cheng Kung University, Tainan, Taiwan; c School of Medicine for International Students, College of Medicine, I-Shou University, Kaohsiung, Taiwan

**Keywords:** Service-learning, medical education, gender differences, empathy development, qualitative analysis

## Abstract

Empathy development is a critical component of medical education, shaping future physicians’ ability to provide compassionate, patient-centered care. This study investigates the reflections of male and female medical students on their experiences at a juvenile correctional school, focusing on how gender shapes their interpretations, emotional engagement, and learning outcomes. Using a qualitative analysis of reflective journals collected from 28 participants, the study employed thematic analysis to identify gender-based patterns in emotional engagement, interpretive framing, and learning outcomes. Reflexivity measures and rigorous coding processes were implemented to ensure credibility and minimize bias in data interpretation. Male students frequently adopted a practical form of empathy, characterized by task-oriented interactions and a sense of competitive camaraderie. In contrast, female students exhibited relational empathy, emphasizing emotional connections and nurturing behaviors. These findings underscore the value of integrating gender-sensitive empathy training into medical education, encouraging the development of both relational and practical approaches. By incorporating tailored reflection exercises and interactive strategies, medical education programs can better equip students to engage compassionately with diverse patient populations. This approach enriches the learning experience and fosters a more holistic understanding of patient care, ultimately leading to improved healthcare outcomes.

## Introduction

Medical education increasingly incorporates experiential learning to foster critical soft skills, including empathy, emotional intelligence, and cultural competence [1]. Visits to juvenile correctional facilities offer medical students unique opportunities to engage with populations outside the clinical environment, challenging their preconceptions and deepening their understanding of societal structures and the role of social support in shaping individual outcomes [2]. Service learning, a key component of medical education, enables students to integrate academic knowledge with hands-on experience in community settings. This approach bridges classroom learning with practical application, promoting holistic development and critical competencies such as social responsibility, empathy, and cultural awareness – essential for effective healthcare practice [3]. Research underscores the positive impact of service learning on academic performance, critical thinking, and writing abilities, fostering personal attributes like leadership, self-efficacy, and a commitment to social justice and civic responsibility. Through reflective practice, service learning not only reinforces course material but also strengthens students’ long-term dedication to community service and professional growth [4].

Service learning is a pivotal component in cultivating cultural competence within medical education, providing students with immersive experiences in settings that require nuanced understanding and respect for diverse cultural norms and practices. Such exposure is essential for fostering patient-centered care skills in increasingly multicultural healthcare systems, where cultural sensitivity enhances patient-provider relationships and contributes to improved health outcomes, particularly among underserved and minority populations. Studies indicate that participation in cross-cultural care programs and service-learning initiatives significantly enriches students’ cultural awareness, communication abilities, and empathy toward diverse patient groups, thereby enhancing their confidence and effectiveness in delivering care across varied cultural contexts [5,6].

Integrating service learning within medical curricula aligns with the fundamental objectives of medical education, which seeks to produce physicians who are not only technically proficient but also socially accountable and committed to the well-being of their communities. As a sophisticated form of experiential learning, service learning offers students direct engagement in health-related initiatives, deepening their insights into the social determinants of health and ethical considerations in patient care [3]. This experiential approach fosters essential competencies in self-awareness, empathy, and reflective practice – key elements for effective patient care and professional development. By navigating cultural complexities and working within diverse settings, medical students acquire both the clinical proficiency and intercultural adaptability necessary to address the demands of a globalized healthcare environment [7].

The School of Medicine for International Students at I-Shou University in Taiwan embodies this educational approach, particularly benefiting international students from Pacific and Caribbean nations. Many of these students, awarded full scholarships, are prepared to enhance public health systems and medical infrastructure in their home countries. A central component of their curriculum is service learning in medical humanities, designed to complement clinical training by immersing students in public welfare initiatives that foster empathy, social responsibility, and cultural competence. A prime example is their visit to a juvenile correctional school in Taiwan on 12 March 2024, where students confronted preconceptions and engaged deeply with issues of social justice, resilience, and the role of structured support in transformative care settings. This hands-on experience prompted critical reflection on societal influences on health, reinforcing their roles as compassionate, culturally sensitive healthcare professionals.

Gendered socialization often shapes empathetic capacities and emotional responses differently in females and males, impacting professional fields such as medicine. Females are frequently socialized to develop nurturing and empathetic qualities, fostering strong capacities for emotional connection and compassionate care, which become particularly prominent in patient-centered roles. Studies consistently find that female medical students score higher on empathy measures, reflecting a readiness for emotionally engaged patient interactions [8,9]. In contrast, males are often socialized toward resilience, independence, and emotional restraint, promoting a more analytical orientation to patient care where empathy integrates with technical proficiency and problem-solving. While capable of empathy, male students often express it through structured analytical responses rather than immediate emotional engagement [10].

Empirical studies within medical education consistently reveal that female medical students score higher on measures of empathy and compassion. This suggests a stronger orientation toward empathetic care among females, who may engage with patients on an emotional level more readily than their male counterparts [8,11]. Male students, while capable of empathy, often express it through problem-solving and analytical frameworks rather than through immediate emotional engagement. Recognizing these gendered influences is crucial for developing balanced medical training programs that foster empathy across genders. Tailoring educational approaches can help equip all medical students to provide compassionate, patient-centered care while respecting diverse emotional development styles [8,10].

This study aimed to analyze gender-based differences in how male and female international medical students perceive, engage, and reflect on empathy, cultural competence, and social responsibility following a service-learning experience in a juvenile correctional school. By examining these interactions, the study sought to understand how students’ perspectives on professional growth and patient care were influenced and to assess the role of service learning in fostering personal development. Additionally, the study explored the implications of gender on how these insights are integrated into clinical practice, highlighting potential differences in approaches to patient care shaped by these formative experiences.

## Methods

### Participants

The study included 28 first- and second-year medical students from the School of Medicine for International Students at I-Shou University. The participants represented both genders and came from diverse nationalities, including Belize and St. Lucia, bringing with them varied linguistic and cultural backgrounds. All participants engaged in a structured service-learning activity conducted on 12 March 2024, at a juvenile correctional facility in Taiwan. During this program, students participated in direct, interactive sessions with juvenile residents. The activity was designed to enhance cross-cultural understanding, foster empathy, and deepen ethical awareness, emphasizing the critical role of social responsibility in shaping future medical practitioners.

The structured environment of the service-learning experience offered a distinctive opportunity to explore reflections on complex social interactions and cultural dynamics, particularly emphasizing gender-based variations in perceptions and responses. The balanced representation of genders among participants facilitated a comparative analysis, providing valuable insights into how male and female students navigated emotionally charged and multifaceted experiences in a non-clinical setting.

### Study design and setting

This study employs a qualitative research design to examine the gender-specific reflections of international medical students following their participation in a service-learning activity at a juvenile correctional facility in Taiwan. Rooted in a service-learning framework, the study aimed to cultivate critical professional competencies essential for future healthcare providers, including social responsibility, empathy, and relational skills. The guided visit offered students a meaningful opportunity to engage with resident juveniles through structured interactions meticulously designed to facilitate observing behaviors, aspirations, and backgrounds within a carefully controlled and supportive environment, fostering experiential learning and reflective practice.

Reflection was integral to the study design, prompting participants to critically evaluate their initial expectations, interactions, and ultimate insights regarding social environments and structural support within the facility. Thematic analysis was employed to systematically identify gendered patterns in students’ reflections, facilitating a nuanced understanding of the subjective experiences shared by male and female students. Findings contribute to discussions on incorporating gender-sensitive approaches in curriculum development, highlighting the role of service learning as a pedagogical strategy in enhancing empathy and relational skills in medical education.

### Reflexivity statement

The research team consisted of experienced medical educators and qualitative researchers with diverse academic and cultural backgrounds. Recognizing the potential influence of personal biases on the study design and data interpretation, reflexivity was maintained throughout the research process. Researchers were mindful of their roles as facilitators of the service-learning program and acknowledged how their positions might shape participants’ reflections and the analysis of those reflections.

To mitigate bias, we implemented several strategies, including independent coding by multiple researchers, iterative discussions to reconcile differing interpretations, and member checking with participants to validate preliminary findings. Additionally, the research team engaged in regular self-reflection to critically examine how their cultural, academic, and professional contexts might influence their understanding of gender dynamics and empathy development. These measures ensured that the findings authentically represented the participants’ voices while maintaining the rigor and trustworthiness of the study.

### Student reflection prompts

To systematically examine students’ evolving perceptions, emotional responses, and professional insights regarding empathy development, patient care, and social determinants of health, a structured reflection exercise was embedded within the study. Participants were required to submit structured reflections following their service-learning visit to the juvenile correctional school. These reflections were analyzed using thematic analysis to identify emergent patterns in empathy development and professional growth.

A set of structured reflection prompts was provided to ensure consistency in data collection and facilitate an in-depth exploration of students’ experiences. These prompts encompassed pre-visit expectations, observational insights, emotional responses, development of empathy, cultural awareness, ethical considerations, and applications to medical practice. The complete set of prompts is detailed in Supplementary data 1.

By integrating a structured approach to qualitative inquiry, this exercise enabled a systematic and rigorous analysis of student reflections. Data were systematically coded, and key themes were iteratively refined through collaborative analysis by multiple researchers, ensuring methodological robustness, reliability, and validity in the study’s findings.

### Data collection

Data were collected through reflective journaling, where medical students submitted detailed written reflections one week after participating in the service-learning activity at the juvenile correctional school. This timeframe was strategically chosen to balance immediacy and reflection, allowing students to thoughtfully process their experiences while maintaining an emotional connection to the event. The reflections were designed to capture a comprehensive range of insights, including students’ initial expectations, observations during interactions with juveniles, perceptions of the facility environment, and reflections on broader social structures and future aspirations. Guided prompts encouraged participants to explore both cognitive and emotional dimensions of their experiences, ensuring the documentation of diverse perspectives shaped by gender, socialization, and cultural background. This qualitative data provided a rich narrative framework, offering deep insights into students’ experiential learning. It illuminated nuanced perceptions, attitudes, and transformative processes influenced by personal experiences and societal factors, contributing to a more holistic understanding of their developmental journey through service learning.

### Data analysis

The qualitative data analysis was conducted using ATLAS.ti, a qualitative data analysis software that facilitates systematic coding, thematic categorization, and comparative analysis. To ensure methodological rigor and reliability, a structured, multi-phase approach was employed, allowing for the identification of gender-based differences in medical students’ reflections on their experiences at the juvenile correctional school. Following best practices in qualitative research, the analysis incorporated multiple coding stages, inter-coder reliability assessments, and iterative refinements to enhance the validity and consistency of findings.

A qualitative thematic analysis was applied to systematically examine students’ reflective journals from their service-learning experiences. Utilizing a structured, multi-stage coding process, the study ensured analytical consistency and validity in identifying key themes. This approach enabled a comprehensive and nuanced exploration of students’ perspectives while minimizing researcher bias and enhancing the credibility of the findings.

#### Step 1. Data familiarization

To develop a deep understanding of students’ reflective narratives, all journal entries were read multiple times. This immersion phase allowed for an initial sense of emerging themes and patterns. Detailed notes were taken to capture preliminary observations, including recurrent motifs, emotional tone, and variations in student perspectives.

#### Step 2. Initial code generation

The data were segmented into discrete meaning units, and each segment was assigned a descriptive code that captured its essential meaning. Open coding was conducted inductively, ensuring that codes emerged directly from the data rather than imposing a priori. This approach facilitated the identification of novel insights specific to students’ lived experiences in the service-learning context.

#### Step 3. Clustering related codes into sub-themes

Following initial code generation, related codes were grouped into higher-order conceptual categories. This process of axial coding enabled the identification of interrelationships between codes, allowing for the emergence of cohesive sub-themes. The clustering of related data points ensured that key aspects of student reflections were not analyzed in isolation but rather contextualized within broader thematic structures.

#### Step 4. Development of thematic framework

The final stage of the coding process involved synthesizing the sub-themes into overarching thematic domains, forming a hierarchical structure that encapsulated the primary findings. The final thematic framework was aligned with the thematic patterns reported in Supplementary data 2.

#### Step 5. Ensuring coding consistency and reliability

To enhance the rigor and trustworthiness of the thematic analysis, several measures were systematically implemented. Investigator triangulation was applied, with two independent researchers conducting the coding process separately, and discrepancies were resolved through consensus discussions to enhance interpretative validity. Iterative code refinement ensured that codes and thematic categories were continuously reviewed and adjusted to accurately represent the full breadth of student experiences, preventing overgeneralization or reductionism. Representative quotes validation was conducted by reviewing selected reflective excerpts to confirm that each identified theme was well-supported by qualitative data, with representative quotes incorporated in the results to illustrate each thematic domain and provide contextual depth. Finally, an audit trail and reflexivity approach were maintained through a systematic record of coding decisions, theme evolution, and researcher reflections, ensuring methodological transparency and mitigating potential biases.

#### Ethical considerations and confidentiality

The study received approval from the Institutional Review Board at I-Shou University (IRB No. 2024025), ensuring adherence to ethical research protocols. Given the sensitive nature of the juvenile correctional school environment, stringent confidentiality measures were implemented to safeguard the privacy of all participants. The school, operating under the Correctional Agency, Ministry of Justice, serves as a specialized educational institution that provides continued formal education to legal offenders. Access to the facility is highly restricted, requiring pre-approved personnel lists, security screenings, and continuous supervision by correctional school staff to maintain institutional security and order.

To uphold strict confidentiality, all forms of recording devices, including cameras, audio recorders, and mobile phones, were prohibited within the facility. Medical students participating in the study were required to leave all electronic devices outside before entering and were accompanied at all times by school personnel to ensure compliance with institutional protocols. Furthermore, all reflective journal entries analyzed in the study were fully anonymized, containing no identifying information about either the medical students or the juvenile residents. No direct quotes or personal details of juvenile residents were recorded or published, ensuring that only the students’ reflections were included in the dataset. These measures align with ethical research guidelines for studies conducted in correctional and educational settings, ensuring data confidentiality and secure handling throughout the research process.

## Results

The age distribution of participating students varied by nationality, years of study, and gender, which is presented in Table 1. First-year female students from Belize had a mean age of 28.0 years, while those from St. Lucia were younger at 23.0 years. First-year male students from St. Lucia had the highest average age at 31.0 years. Among second-year students, gender disparities in age were less pronounced for Belizean participants (females: 25.4 years, males: 28.0 years), while St. Lucian students maintained a higher age average (females: 27.4 years, males: 32.0 years).

**Table 1.** Age distribution by school year, nationality, and gender.

Year	Nationality	Gender	Count	Mean age	SD
First	Belize	Female	1	28	
First	St. Lucia	Female	6	23	2.8
First	St. Lucia	Male	2	31	4.2
First	St. Vincent & the Grenadines	Female	1	25	
First	Tuvalu	Male	1	31	
Second	Belize	Female	5	25.4	1.7
Second	Belize	Male	3	28	3
Second	St. Lucia	Female	8	27.4	4.7
Second	St. Lucia	Male	1	32	

### Gender differences analysis

Analysis of student reflections reveals key gender-based themes related to initial expectations, interactions with juveniles, perceptions of kindness and optimism, and reflections on future aspirations (Table 2). These findings illustrate how socialization influences engagement styles, emotional responses, and perspectives in non-clinical settings.

**Table 2.** Summary of gender differences in key themes in medical students’ reflections on juvenile correctional school visits.

Theme	Male students	Female students
Initial expectations and reactions	Expressed apprehension and concerns about violence; framed by societal stereotypes and discussions with peers, adopting a cautious, defensive stance.	Approached with empathy, focusing on the potential for change, reflecting a nurturing stance focused on understanding individuals beyond the ‘offender’ label.
Interactions with students	Focused on physical interactions like arm-wrestling, reflecting traditional masculine behaviors and building camaraderie.	Emphasized emotional connections, focusing on juveniles’ creativity, resilience, and relational dynamics over competition.
Perceptions of kindness and optimism	Viewed kindness as individual actions rather than as products of the environment, highlighting a more compartmentalized perception of juveniles’ positive attributes.	Recognized the positive, supportive environment created by school staff, framing kindness and optimism within the context of a nurturing setting that fosters growth and resilience.
Future aspirations and social awareness	Reflected on social structures and privilege, recognizing the narrow divide between their own lives and those of the juveniles, prompting introspection about societal influences on life trajectories.	Emphasized transformation and second chances, believing in the power of community support to positively alter at-risk youths’ futures; framed narratives within a broader context of collaborative problem-solving and social support.

#### Initial expectations and reactions

Male students commonly expressed apprehension and caution, shaped by societal narratives surrounding juvenile offenders. Concerns about personal safety and potential violence were predominant themes, reinforced by peer discussions that framed juveniles within a criminalized context. This defensive stance reflects broader gendered socialization patterns, wherein males are often encouraged to approach unfamiliar social environments with emotional restraint and caution.

Conversely, female students exhibited a more open and empathetic perspective, with reflections emphasizing the humanity of the juveniles rather than their legal status. They were less concerned with safety and more inclined to explore the juveniles’ personal histories and rehabilitative potential. This relational orientation aligns with prior research suggesting that women are more likely to engage empathetically and express curiosity about individual experiences in caregiving contexts.

#### Interactions with juvenile students

Differences in engagement styles were evident during interactions at the correctional facility.

Male students gravitated toward physical engagement, such as arm-wrestling and competitive activities, which served as mechanisms for building camaraderie and gradually challenging preconceived stereotypes. These interactions facilitated peer-like connections, shifting perceptions of juveniles from outsiders to relatable individuals.

Female students prioritized emotionally driven interactions, focusing on the juveniles’ creativity, intelligence, and resilience. Their reflections emphasized personal conversations, relational insights, and expressions of support, reinforcing an emotionally attuned approach to engagement.

These gendered engagement styles highlight distinct pathways to empathy development, with males using shared physical activities to bridge social gaps, while females focused on establishing interpersonal connections through dialogue and emotional understanding.

#### Perceptions of kindness and optimism

Both male and female students recognized the juveniles’ kindness and optimism, yet they interpreted these traits differently.

Male students viewed acts of kindness as individual behaviors, often isolated from broader environmental influences. Their reflections emphasized specific gestures of friendliness but did not frequently link them to the correctional facility’s structured rehabilitative setting.

Female students contextualized kindness within the broader institutional environment, crediting supportive staff and rehabilitative frameworks for fostering a positive social atmosphere. Their reflections suggest a holistic interpretation of kindness, recognizing the interplay between structured support and individual behavior.

This difference reflects cognitive processing tendencies, wherein males exhibited a more compartmentalized perspective, whereas females considered relational and contextual influences on social behavior.

#### Reflections on future aspirations and social structure awareness

Reflections on the juveniles’ aspirations revealed distinct gender-based analytical approaches:

Male students engaged in introspective and structural analyses, often drawing parallels between their own life trajectories and those of the juveniles. Their reflections frequently acknowledged systemic inequalities, privilege, and social determinants influencing life outcomes.

Female students emphasized individual transformation and the role of community support in rehabilitation. Their reflections framed the juveniles’ potential for change within collaborative networks, reinforcing optimism about rehabilitation and second chances.

This contrast suggests that male students approached aspirations from a systemic perspective, whereas female students viewed rehabilitation through a more relational and community-oriented lens.

### Thematic analysis of gender-based insights

Beyond gender-specific reflections, overarching themes emerged across both groups (Tables 3 and 4). These themes provide a structured framework for understanding students’ engagement, empathy development, and key takeaways from the service-learning experience.

**Table 3.** Thematic analysis of student reflections with coded dialogues on juvenile correctional school visits.

Code item	Theme description	Example dialogue
Empathy	Reflects the ability to understand and share the feelings of others, particularly in the context of interactions with juvenile students.	‘I could see how much they wanted to be understood.’
Compassion	Highlights the caring and supportive attitudes exhibited by medical students towards the juvenile population.	‘It broke my heart to see them struggle, but I wanted to help.’
Transformation	Represents the changes observed in students’ behaviors and attitudes as a result of the correctional program.	‘I noticed how some of them have really turned their lives around.’
Positive change	Indicates the improvements in the lives and outlooks of juvenile students, emphasizing hope and growth.	‘They have so much potential; it’s inspiring to see their progress.’
Student participation	Captures the active involvement of both medical and juvenile students during activities and interactions.	‘Everyone was eager to join in and share their stories.’
Active engagement	Reflects the dynamic interactions and enthusiasm displayed by students during the visit.	‘The energy in the room was contagious; we were all laughing and having fun.’
Future aspirations	Represents the hopes and goals expressed by juvenile students regarding their futures and potential for change.	‘I want to be a doctor one day; I know I can do it.’
Nurturing environment	Describes the supportive and caring atmosphere of the correctional school that fosters positive interactions.	‘The teachers really care about us; it makes a difference.’
Resilience	Highlights the ability of juvenile students to overcome challenges and maintain hope despite their circumstances.	‘Even after everything, I still believe I can change my life.’
Emotional connections	Reflects the bonds formed between medical students and juvenile students, emphasizing relational dynamics.	‘I felt a real connection with them; it was like we understood each other.’

**Table 4.** The frequency and description of each theme in student reflections.

Theme	Frequency	Description
Empathy	8	The ability to understand and share the feelings of others, particularly in interactions with juvenile students.
Compassion	6	The caring and supportive attitudes exhibited by medical students towards the juvenile population.
Transformation	7	The changes observed in students’ behaviors and attitudes as a result of the correctional program.
Positive change	5	Improvements in the lives and outlooks of juvenile students, emphasizing hope and growth.
Student participation	6	The active involvement of both medical and juvenile students during activities and interactions.
Active engagement	7	The dynamic interactions and enthusiasm displayed by students during the visit.
Future aspirations	5	The hopes and goals expressed by juvenile students regarding their futures and potential for change.
Nurturing environment	6	The supportive and caring atmosphere of the correctional school fosters positive interactions.
Resilience	4	The ability of juvenile students to overcome challenges and maintain hope despite their circumstances.
Emotional connections	5	The bonds formed between medical students and juvenile students, emphasize relational dynamics.

#### Empathy and compassion

Female students frequently exhibited a predisposition for understanding and openness, while male students developed empathy through structured engagement and observation.

#### Transformation and positive change

Both genders recognized the facility’s rehabilitative impact, with males emphasizing behavioral changes and females focusing on emotional resilience.

#### Active participation and engagement

Male students favored physical activities, while female students engaged in meaningful dialogues to form deeper personal connections.

#### Future aspirations and social structure awareness

Male students reflected on structural inequalities, while female students emphasized mentorship and the importance of community networks in facilitating rehabilitation.

#### Resilience and emotional connections

Both groups admired the juveniles’ perseverance, with females emphasizing emotional strength and males recognizing determination and effort.

## Discussion

The thematic analysis of medical students’ reflections on their visit to a juvenile correctional facility reveals significant gender-based distinctions in perception, emotional engagement, and interpretive framing. These insights underscore how gendered socialization patterns, embedded within broader cultural narratives, influence students’ approaches to understanding and engaging with marginalized and vulnerable populations. Through thematic constructs – empathy, resilience, structural awareness, emotional connection, and perceptions of a nurturing environment – it becomes evident that male and female students processed their experiences differently. Male students generally adopted a cautious, analytical stance, emphasizing resilience and structural factors affecting life trajectories. Conversely, female students displayed a more empathetic and optimistic perspective, underscoring emotional connection and the importance of nurturing environments, envisioning pathways for transformation through sustained community support. These findings not only highlight the influence of gender socialization on interpreting complex social contexts but also suggest an avenue for developing balanced, complementary competencies in empathy, resilience, and systems-based understanding within clinical settings.

Beyond gender differences, this study underscores the pivotal role of service-learning in shaping medical students’ professional development and perspectives on patient care. Through direct engagement with juvenile residents, students gained firsthand exposure to the complexities of social determinants of health and the critical role of empathy in clinical practice. Reflective journal analyses revealed that while many students initially approached healthcare with a predominantly clinical perspective, their experiences at the juvenile correctional school prompted a paradigm shift, emphasizing the necessity of integrating social awareness into patient interactions [12]. Several students articulated a heightened understanding of the multifaceted challenges faced by underserved populations, recognizing that comprehensive patient care extends beyond medical interventions to encompass emotional and social support. This transformation suggests that service-learning experiences reinforce clinical competencies and cultivate essential relational and ethical skills, fostering a more holistic and patient-centered approach to care [13]. Integrating such experiential learning opportunities within medical curricula may enhance students’ capacity to engage effectively with diverse patient populations, ultimately contributing to more compassionate and socially responsive medical practice [14,15].

### Gender socialization and initial expectations

The reflections indicate that gender socialization plays a foundational role in shaping students’ initial expectations and emotional responses toward marginalized groups, particularly in the context of a juvenile correctional facility, where preconceptions about ‘juvenile offenders’ may be heightened [16]. Male students’ reflections often revealed a cautious approach marked by apprehension, rooted in societal narratives that portray juvenile offenders as potentially dangerous. This cautious stance reflects a protective response, influenced by cultural expectations that males prioritize self-protection and maintain emotional distance in ambiguous or challenging settings. Their reflections demonstrate a focus on structural factors shaping the juveniles’ lives, hinting at a preference for understanding behavior through a systemic lens rather than through individual narratives. Such an approach aligns with research showing that males may adopt a more detached, system-oriented view in complex social situations, prioritizing analyses of socioeconomic determinants, environmental stressors, and institutional structures that affect individual behaviors [17]. This stance on social determinants not only reflects an analytical frame but may also suggest a tendency to internalize societal expectations around masculinity, potentially limiting emotional engagement in ambiguous environments.

On the other hand, female students approached the experience with a relational focus, marked by a readiness to engage emotionally with the juveniles and a curiosity to understand them beyond the ‘offender’ label. This open, empathetic stance reflects a socialization pattern that encourages women to adopt nurturing and supportive roles in interpersonal contexts. The reflections from female students frequently highlight their interest in understanding the juveniles’ life experiences, revealing a propensity to see beyond immediate behavior to the broader potential for growth and change. This inclination aligns with studies suggesting that females are often socialized to emphasize empathy, compassion, and supportive interactions in emotionally charged settings and people-oriented specialty [18]. This difference in initial expectations underscores the importance of acknowledging how socialization influences students’ approaches in diverse, non-clinical settings and contributes to a foundation of varied, yet complementary, professional competencies in empathy and patient-centered care [19]. Recognizing these gendered perspectives within medical education offers a potential framework for developing balanced emotional engagement and resilience in clinical interactions.

### Gendered interaction styles with juveniles

Distinct gender-based interaction styles were evident during the students’ engagement with the juveniles, underscoring the influence of socialization on interpersonal behaviors. Male students primarily engaged through physical activities, such as arm-wrestling, a traditional form of masculine bonding marked by competition and assertiveness. This physical engagement provided a means to break down initial stereotypes, enabling the male students to transition from perceiving the juveniles as ‘others’ to seeing them as peers. These competitive, activity-based interactions allowed male students to build camaraderie and offered a non-verbal means of connection, which may serve as a bridge for emotional understanding in future clinical interactions. This type of engagement reflects research suggesting that men often bond through shared physical or competitive activities, fostering connections through action rather than emotional exchange.

In contrast, female students prioritized emotionally connective interactions, focusing on the juveniles’ creativity, resilience, and potential for growth. Their approach emphasizes relational engagement, allowing for deeper connections that recognize juveniles as individuals with intrinsic worth and potential. This tendency aligns with literature highlighting the role of emotional intelligence and empathy in female socialization, promoting sensitivity to others’ needs and fostering supportive interactions. The emphasis on relational engagement may indicate an increased capacity to form therapeutic alliances in clinical contexts, suggesting that female students may prioritize understanding patients’ emotional and psychological landscapes as integral to medical care [19]. This nuanced difference in interaction styles underscores the importance of cultivating diverse interpersonal approaches within medical education, acknowledging that both action-based and emotionally connective strategies contribute to holistic patient care.

### Differing perceptions of kindness and optimism

Both male and female students noted the kindness and optimism exhibited by the juveniles; however, their interpretations diverged, reflecting broader patterns in gendered perception. Male students often perceived acts of kindness as isolated behaviors rather than as reflections of the broader supportive environment, suggesting a compartmentalized view that frames positive behaviors within structural or systemic contexts. This tendency toward analytical compartmentalization may be rooted in a desire to understand individual actions within a larger socio-economic and institutional framework, rather than situating them within an immediate, nurturing context. This outlook could serve as a foundation for assessing external determinants of behavior within clinical care, focusing on the impact of social, environmental, and economic factors on health outcomes [20–22].

Female students, however, were more likely to view these positive behaviors as a direct outcome of the emotionally supportive environment created by school staff, highlighting the importance of contextual, social support systems in shaping attitudes and behaviors. This relational interpretation aligns with findings suggesting that females are more attuned to the social context surrounding individual behaviors, often viewing kindness and optimism as products of a nurturing environment [20,23]. This perspective is significant in medical education as it reinforces the relevance of integrating socio-emotional support into healthcare, promoting a comprehensive understanding of how environmental factors and interpersonal relationships contribute to patient well-being [21,22].

### Reflections on aspirations and structural determinants

Students’ reflections on the juveniles’ future aspirations and life circumstances further reveal gender-based distinctions in outlook and interpretative frameworks. Male students frequently engaged in reflective analysis on the impact of structural inequities, such as socio-economic disparities, on youth outcomes. This analytical approach suggests that male students may possess an inclination toward examining systemic factors that shape life trajectories, emphasizing resilience as an external challenge influenced by social determinants. Such perspectives resonate with an approach in healthcare that considers structural and systemic influences on patient outcomes, fostering a framework where resilience is viewed as a response to external adversities [21]. By contemplating the broader social structures that influence individual lives, male students demonstrate an interest in understanding resilience as a process of navigating systemic barriers, which may inform their future role in advocating for health equity [20].

Female students, by contrast, expressed an optimistic outlook regarding the potential for transformation in the juveniles’ lives, focusing on the importance of community support and a nurturing environment as catalysts for positive change. This perspective aligns with the salutogenic model [24], which emphasizes the creation of health and wellbeing through supportive social environments [22,24]. Female students’ reflections on the potential of supportive networks to foster resilience and self-growth highlight an approach that centers on the psychosocial aspects of health. This orientation towards community-centered frameworks in healthcare underscores the potential of medical education to instill values of social support, empathy, and relational engagement, which are essential for fostering resilience and hope in clinical practice [20]. By appreciating the transformative potential of nurturing environments, female students bring forward a model of care that emphasizes patient-centered support as foundational to effective medical intervention [21].

### Empathy and compassion: gender differences in approach

Empathy and compassion emerged as prominent themes in the reflections, with notable variations between male and female students’ approaches to these qualities. Female students’ reflections tended to demonstrate a deep, individualized empathy for the juveniles, framing their observations around understanding each juvenile’s unique story and potential for change. This aligns with research indicating that females, through socialization, are often encouraged to adopt relational and nurturing approaches, which emphasize emotional connection and a supportive stance [8,25]. In medical education, studies have shown that female students often score higher on measures of empathic concern, suggesting a tendency to value compassionate, patient-centered care [25]. Female students’ reflections were rich in emotionally supportive language, frequently expressing admiration for the juveniles’ resilience and a desire to contribute positively to their lives [10]. This empathetic orientation aligns with studies showing that female physicians and medical students often prioritize interpersonal aspects of patient care, viewing empathy as central to professional competence [26,27].

### Cognitive and structural dimensions of empathy in male students

The reflections of male students on empathy reveal a distinct analytical approach, contextualizing their understanding of juvenile behavior within broader structural and societal frameworks. This perspective emphasizes a cognitive dimension to empathy, where male students consider the social determinants of juvenile delinquency and the precarious life choices leading to incarceration. Research indicates that male students often engage with empathy, including cognitive and emotive components [28], from a cognitive standpoint, prioritizing an understanding of circumstances over immediate emotional responses [29]. This cognitive emphasis aligns with findings indicating that males may exhibit lower levels of emotional empathy compared to females, focusing instead on structural insights related to socioeconomic disparities, family dynamics, and educational inequities affecting juveniles.

By highlighting these structural factors, male students demonstrate an awareness of the broader societal influences on juvenile behavior. This analytical lens serves as a valuable complement to emotional empathy, fostering a more holistic view of patient backgrounds and societal influences, which is crucial for culturally competent medical care. Empathy in this context not only aids in understanding individual circumstances but also promotes a comprehensive approach to addressing the challenges faced by at-risk populations. Incorporating both cognitive and emotional dimensions of empathy into medical education can enhance future physicians’ abilities to provide compassionate care while recognizing the complex social contexts that shape their patients’ lives [28].

### Resilience: gender perspectives on strength and adaptability

Resilience was another salient theme across reflections, with male and female students demonstrating differing emphases on this quality in the juveniles. The reflections on resilience among medical students reveal significant gender differences in how this quality is perceived and emphasized [30,31]. Male students often focus on observable behaviors and physical resilience, viewing these traits as indicators of potential for adaptation and improvement [32]. This aligns with traditional masculine socialization, which values resilience as a form of behavioral endurance [25]. Male students expressed admiration for juveniles who engaged in challenging activities, interpreting their determination as a sign of strength and fostering a sense of camaraderie among peers.

Conversely, female students framed resilience through an emotional and relational lens, emphasizing inner strength and perseverance despite adversity [25]. They highlighted emotional growth and self-reflection, suggesting that females may be more attuned to resilience as an internal quality linked to psychological adaptability. This perspective reflects a broader understanding of resilience that includes emotional fortitude alongside behavioral adaptation, acknowledging the juveniles’ ability to navigate hardships while maintaining hope for transformation. Research supports these observations, indicating that fostering a comprehensive understanding of resilience in medical education is crucial [25]. It encourages future physicians to recognize and support both patients’ psychological and physical resilience, ultimately enhancing the overall well-being of medical professionals and their patients.

### Implications for medical education

This study underscores the importance of integrating service learning into medical curricula to foster both analytical and empathetic competencies that are crucial for navigating the complexities of patient care in diverse social contexts. Service-learning experiences, especially in non-clinical environments like juvenile correctional facilities, offer medical students invaluable opportunities to deepen their understanding of social determinants of health, practice empathy, and engage in complex social settings [33,34]. By embedding structured reflection on gender perspectives, empathy, and social determinants within these experiences, medical education programs can enhance students’ emotional intelligence, cultural competence, and readiness to work with patients from varied backgrounds.

The findings highlight the value of encouraging future physicians to recognize both individual and systemic factors impacting health while fostering empathy and resilience, ultimately strengthening their capacity for patient-centered care. Reflective exercises that allow students to explore how gender may shape their approach to empathy and resilience further promote self-awareness and adaptability, equipping both male and female students to connect meaningfully with a broad spectrum of patients [35]. Acknowledging gender-based differences in processing social influences can help inform training programs, ensuring they support diverse engagement styles with vulnerable populations.

As healthcare shifts increasingly toward patient-centered models, medical curricula that emphasize relational awareness, structural insights, and compassion are critical for preparing students to address patient needs holistically and ethically. Future studies could build on these insights by exploring how service-learning across varied cultural and institutional contexts influences empathy, resilience, and professional identity, offering a broader perspective on the impact of socialization and gender in medical education.

## Limitations

This study has several limitations that should be considered when interpreting its findings. First, the sample consisted of international medical students from specific national backgrounds, which may limit the generalizability of the results. While participants came from diverse backgrounds, they were studying within an Eastern cultural context, where variations in educational systems, societal norms, and gender role expectations may have influenced their reflections. These cultural factors shaped students’ perspectives on empathy, resilience, and structural awareness, as well as their interpretation of service-learning experiences. Consequently, differences in how male and female students engaged with juvenile residents and processed their experiences may, in part, be attributed to these cultural dynamics.

Second, although this study primarily focused on gender-based differences in student reflections, we acknowledge that other individual background factors – including cultural upbringing, educational history, personal experiences, and socio-economic status – may have influenced participants’ perceptions and responses. While our post-hoc analysis did not reveal significant differences based on age, we recognize that varying levels of social experience may shape students’ engagement and reflection. Future research with a larger sample and more comprehensive demographic data could provide a deeper understanding of these potential influences.

Furthermore, this study did not collect extensive background information – such as childhood experiences, prior exposure to similar social settings, or personal histories of adversity – due to ethical considerations and the need to maintain participant confidentiality. While we acknowledge that past experiences, including adverse childhood events, may shape empathy development, all participants were encountering the juvenile correctional school environment for the first time. Their reflections, therefore, primarily captured their immediate engagement rather than prior familiarity with such settings. To further elucidate how personal history influences empathy development, future studies could integrate more detailed demographic and psychological assessments while ensuring strict ethical safeguards.

Another limitation is the reliance on self-reported reflections, which are inherently subject to social desirability bias. Given the sensitive nature of working in a correctional facility, students may have felt compelled to align their responses with perceived expectations regarding empathy and professionalism, despite assurances of confidentiality. The absence of objective measures to validate these self-reported reflections may limit the study’s findings. Incorporating observational data, peer assessments, or faculty evaluations in future research could provide a more holistic perspective for students’ engagement and learning.

Additionally, reflections were collected immediately following the service-learning experience, capturing primarily short-term emotional responses rather than sustained changes in attitudes or perspectives. While these reflections offer valuable insights into students’ initial reactions, they may not fully represent the long-term impact of the experience. A longitudinal study design with follow-up reflections at later intervals would allow researchers to assess whether the empathy, structural awareness, and professional insights gained are retained over time.

The variability in the quality and depth of student reflections also presents a challenge. Personal introspective ability and comfort with reflection practices can differ significantly between individuals, resulting in varied depth and quality of reflections. Some students may naturally engage more deeply in self-reflection, while others may struggle to articulate their thoughts and emotions in writing. This variability could affect the consistency of the findings, suggesting that future studies could benefit from standardized reflection prompts, guided reflection sessions, or semi-structured interviews to enhance data uniformity.

Moreover, while this study focused on gender-based differences, it may have overlooked other important thematic dimensions, such as ethical considerations, broader professional development, and patient-centered care principles, which are also crucial in medical education. Expanding the analysis to include these dimensions would provide a more comprehensive view of how service-learning experiences contribute to professional growth, beyond gender-specific insights. Additionally, the study relied on a single service-learning experience in a correctional setting, which may not fully represent the diversity of situations that could influence empathy and structural awareness. Future research might explore how different service-learning environments, such as community health settings or public welfare agencies, affect students’ reflections and learning outcomes.

To enhance the practical applicability of these findings, future research should employ a multi-method approach that triangulates data to improve validity and reliability. The integration of observational data, peer assessments, and semi-structured interviews alongside reflective journals would facilitate a more comprehensive evaluation of student engagement, learning outcomes, and behavioral transformations. Furthermore, adopting a longitudinal study design to track students over time would yield deeper insights into the enduring impact of service-learning on empathy, cultural competence, and professional development. Such an approach would allow for a systematic examination of how experiential learning shapes medical students’ attitudes, behaviors, and professional identities across various stages of their education, ultimately informing evidence-based strategies for the sustained and structured incorporation of service-learning into medical curricula.

From an implementation perspective, integrating service-learning into medical education necessitates a carefully structured curriculum that balances experiential learning with conventional academic instruction. A primary challenge involves ensuring logistical feasibility, particularly in establishing and maintaining partnerships with community organizations such as correctional facilities or underserved healthcare settings. Additionally, faculty preparedness is critical, as educators responsible for facilitating these experiences require specialized training in reflective practice, debriefing techniques, and the management of ethically sensitive discussions. To optimize educational outcomes, structured reflection exercises and formative assessments should be embedded within the curriculum to support students in translating their experiences into meaningful professional growth. Strategies such as interdisciplinary collaboration, faculty development initiatives, and the incorporation of digital reflection platforms can further strengthen the systematic and sustainable integration of service-learning, thereby fostering the development of socially responsible and culturally competent healthcare professionals.

## Conclusion

This study elucidates the role of gender in shaping medical students’ perceptions, responses, and reflective processes during a service-learning experience within a juvenile correctional facility. Through detailed thematic analysis, it was observed that female students predominantly engaged with an empathetic, relational approach, while male students exhibited an analytical, structure-focused orientation. These findings highlight the capacity of service-learning to cultivate both relational and analytical skills critical to medical practice. Such immersive experiences enhance students’ understanding of diverse social environments, foster empathy in patient care, and promote critical engagement with broader social determinants of health, contributing to a more comprehensive, socially attuned medical education. Future research could further investigate the effects of similar service-learning programs across varied cultural and institutional settings to elucidate how diverse social and environmental factors shape empathy and professional growth among medical students.

## Supplementary Material

Supplementary_data_Clean.docx
