# Supplementary material for: Exploring gender differences in empathy development among medical students: a qualitative analysis of reflections on juvenile correctional school visits
Source: Med Educ Online. 2025 May 4;30(1):2500556. doi: 10.1080/10872981.2025.2500556 (PMC12054561; doi:10.1080/10872981.2025.2500556)
Supplement: Supplementary_data_Clean.docx [file ZMEO_A_2500556_SM6070.docx]

**Supplementary data 1.** Reflection prompts for student participants

**Introduction to Reflection Exercise**

As part of the qualitative analysis, students were required to submit structured reflections on their service-learning experience at the juvenile correctional school. The purpose of this exercise was to explore their evolving perceptions, emotional responses, and professional insights regarding empathy development, patient care, and the role of social determinants in healthcare.

The following structured reflection prompts guided participants in documenting their experiences:

**1. Pre-visit Expectations and Perceptions**

- What were your initial thoughts and expectations before visiting the juvenile correctional school?
- Did you have any apprehensions or concerns about interacting with the students? If so, what were they?
- What prior knowledge or experiences shaped your perceptions of individuals in correctional facilities?

**2. Observations and Emotional Responses During the Visit**

- Describe the environment of the correctional school. Were there any aspects that stood out to you?
- How did the students respond to your presence and activities?
- Were there any moments that surprised you or challenged your initial assumptions?

**3. Empathy and Personal Growth**

- How did this experience influence your understanding of empathy in healthcare?
- Can you describe a specific interaction or conversation that had a profound impact on you?
- In what ways did this visit shape your perspective on the social and emotional needs of patients from marginalized backgrounds?

**4. Social and Cultural Insights**

- How did the students’ backgrounds, experiences, or aspirations influence your reflections?
- How do you think social determinants, such as family background, education, or socioeconomic status, impact their personal development?
- Did this experience challenge any of your preconceptions about youth in correctional settings?

**5. Professional and Ethical Considerations**

- What ethical considerations arose during your interactions with the students?
- How do you think healthcare professionals can better support vulnerable populations, such as individuals in correctional institutions?
- What role do service-learning experiences play in fostering cultural competence and empathy among medical students?

**6. Post-visit Reflections and Application to Medical Practice**

- How has this experience influenced your understanding of social responsibility in medicine?
- What key takeaways from this visit will you carry forward into your future medical practice?
- If given the opportunity, what aspects of the program would you suggest improving or expanding?

**Supplementary data 2.** Hierarchical structure of thematic categories

| **Category** | **Sub-category** | **Description** |
| --- | --- | --- |
| Emotional and relational growth | Empathy | Understanding and sharing others' emotions |
|  | Compassion | Sensitivity to others' suffering and willingness to help |
|  | Emotional connections | Forming meaningful bonds through shared experiences |
| Personal and professional development | Transformation | Significant changes in perspectives or behaviors |
|  | Positive change | Growth in personal or professional capacities |
|  | Future aspirations | Evolving career goals and long-term motivation |
| Student engagement in service learning | Student participation | Active involvement in service-learning activities |
|  | Active engagement | Sustained commitment and meaningful interaction |
| Environmental and structural influences | Nurturing environment | Supportive conditions that facilitate learning |
|  | Resilience | Adaptability and perseverance in challenging contexts |
